# Impacts of protein quantity and distribution on body composition

**DOI:** 10.3389/fnut.2024.1388986

**Published:** 2024-05-03

**Authors:** Donald K. Layman

**Affiliations:** Department of Food Science and Human Nutrition, University of Illinois at Urbana-Champaign, Urbana, IL, United States

**Keywords:** leucine, muscle mass, muscle protein synthesis, protein requirements, sarcopenia

## Abstract

The importance of meal distribution of dietary protein to optimize muscle mass and body remains unclear, and the findings are intertwined with age, physical activity, and the total quantity and quality of protein consumed. The concept of meal distribution evolved from multiple discoveries about regulating protein synthesis in skeletal muscle. The most significant was the discovery of the role of the branched-chain amino acid leucine as a metabolic signal to initiate a post-meal anabolic period of muscle protein synthesis (MPS) in older adults. Aging is often characterized by loss of muscle mass and function associated with a decline in protein synthesis. The age-related changes in protein synthesis and subsequent muscle atrophy were generally considered inevitable until the discovery of the unique role of leucine for the activation of the mTOR signal complex for the initiation of MPS. Clinical studies demonstrated that older adults (>60 years) require meals with at least 2.8 g of leucine (~30 g of protein) to stimulate MPS. This meal requirement for leucine is not observed in younger adults (<30 years), who produce a nearly linear response of MPS in proportion to the protein content of a meal. These findings suggest that while the efficiency of dietary protein to stimulate MPS declines with aging, the capacity for MPS to respond is maintained if a meal provides adequate protein. While the meal response of MPS to total protein and leucine is established, the long-term impact on muscle mass and body composition remains less clear, at least in part, because the rate of change in muscle mass with aging is small. Because direct diet studies for meal distribution during aging are impractical, research groups have applied meal distribution and the leucine threshold to protein-sparing concepts during acute catabolic conditions such as weight loss. These studies demonstrate enhanced MPS at the first meal after an overnight fast and net sparing of lean body mass during weight loss. While the anabolic benefits of increased protein at the first meal to stimulate MPS are clear, the benefits to long-term changes in muscle mass and body composition in aging adults remain speculative.

## Introduction

The meal distribution of dietary protein is thought to have a positive impact on body composition and skeletal muscle mass; however, outcomes are influenced by age, physical activity, and the quantity and quality of the protein consumed. In general, the total quantity of protein consumed each day appears to be the most important dietary factor affecting lean body mass (1). If quantity is high, the relative importance of quality and meal distribution is likely minimal. However, with an increasingly older population, epidemic health problems of obesity and diabetes, and dietary guidelines shifting toward more plant-based diets, the combined impact of protein quantity, quality, and meal distribution may have increased importance to maintaining healthy skeletal muscles (1–3). This review provides a summary of the data supporting the hypothesis for meal distribution and addresses the limitations of current knowledge.

## Recognizing metabolic roles of amino acids

In large part, the meal distribution hypothesis arises from the discovery of the role of the branched-chain amino acid leucine in the regulation of muscle protein synthesis (MPS). Post-meal changes in plasma and intracellular leucine concentrations serve as a unique meal-related signal for triggering MPS. While all amino acids have a fundamental role as substrates for protein synthesis, each amino acid, and certainly each of the 9 essential amino acids (EAA), has a metabolic role beyond the fundamental role as a building block for new proteins (4). Examples include tryptophan as a precursor to serotonin, methionine and cysteine as precursors to glutathione and taurine, threonine as a substrate for the production of mucin, lysine essential for the synthesis of carnitine, and leucine for the activation of mTORC1 for triggering MPS. For each of these metabolic roles, the pathway is driven by substrate availability and specifically the intracellular amino acid concentration.

Currently, the unique metabolic roles of each of the 9 EAA are often obscured by the use of the generic concept of dietary “protein.” Protein represents a food source for the delivery of EAA. Protein is somewhat like a vitamin pill. There is no requirement for the pill, but there are requirements for each of the essential vitamins inside the pill. Similarly, protein is simply a food structure that delivers amino acids to the digestive tract. We recently suggested a new framework for evaluating the dietary impact of protein by shifting the focus to the individual nutrient requirements for each of the 9 EAA (5). This approach, called the “EAA-9 Equivalence,” provides a transparent and additive framework for evaluating diet quality and optimizing personal nutrition.

## Discoveries supporting meal distribution

There have been three critical discoveries that have modified our understanding of adult protein needs and led to new concepts about the importance of meal distribution. (1) The first discovery involved elucidating the role of the branched-chain amino acid leucine in regulating the meal response of MPS. The discovery of the regulatory role for leucine highlights the difference between the minimum protein required to provide amino acids as building blocks for new proteins versus an optimal protein intake for metabolic roles. (2) The second discovery was that aging results in a decreased response of MPS to a protein meal but that the age-related decline in efficiency could be overcome by increasing the EAA content of individual meals. (3) The third discovery was the finding that the post-meal anabolic response of MPS has a finite duration of 2 to 3 h, suggesting that a single large protein meal (i.e., dinner) might not be the optimal protein distribution for older adults.

### Muscle protein synthesis responds to meal content of leucine in adults

In the 1970s, multiple investigators provided *in vitro* evidence that among all amino acids, leucine had a unique potential to stimulate MPS (6–8). Using isolated diaphragm muscle or the perfused hemi-corpus, these investigators demonstrated that leucine could stimulate protein synthesis in fasted rats, and the response was associated with increased activation of ribosomes (i.e., binding of ribosomes to mRNA), the cellular structures for assembling amino acids for creating new proteins.

Regulation of protein synthesis is complex, but on a macro-level, it can be viewed at two distinct stages: transcription and translation. Transcription reflects gene expression and long-term regulation of the capacity for protein synthesis by controlling the amounts of ribosomes, mRNA, tRNA, and enzymes, while translation reflects short-term regulations of protein synthesis primarily through regulation of proteins called initiation factors that control the activity or efficiency of the protein synthesis machinery (i.e., ribosomes, mRNA, and tRNA).

To test the specific effects of leucine on transcription versus translation, the research group at the University of Illinois conducted an experiment examining muscle protein synthesis with different lengths of food deprivation, including fed, 24-h fasted, and 72-h fasted treatment groups (9). The hypothesis was that leucine would have the greatest effects during short-term food restriction, reflecting regulation at the translation stage, while prolonged starvation would impact transcription and reduce the potential of leucine to stimulate MPS. Consistent with the hypothesis, leucine exhibited the greatest stimulation of MPS in the 24-h fasted animals with minimal to no effect after 72 h. These findings provided evidence that the anabolic effects of leucine were at the initiation stage of MPS and reflected metabolic regulations for recovery after a short-term catabolic period (i.e., in this case, food restriction). This aspect was an early indication that the composition of a meal could alter the rate of MPS.

Proof for the mechanism would wait for more than a decade to develop an antibody methodology for quantitative analysis of the proteins involved in initiation. In collaboration with colleagues at Penn State University, we demonstrated that MPS recovery after an acute catabolic period was regulated in large part by the eIF4 initiation complex (i.e., eIF4E and eIF4G), which is a key regulatory factor for the activation of mRNA and stimulation of MPS (10). Using exhausted exercise to generate an acute catabolic condition, we found that MPS was depressed by over 30% from the pre-exercise stage. Furthermore, this inhibition of MPS was produced by binding an inhibitory protein, binding protein 1 (BP1), to the eIF4E subunit of the eIF4 complex, creating an inactive complex. We showed that the BP1 binding could be reversed within an hour of feeding protein, allowing for eIF4E and eIF4G to bind together and creating the active eIF4 initiation complex for the stimulation of MPS. Subsequently, we demonstrated that eIF4 activation was dependent on the cell concentration of leucine (11).

**Figure 1:**
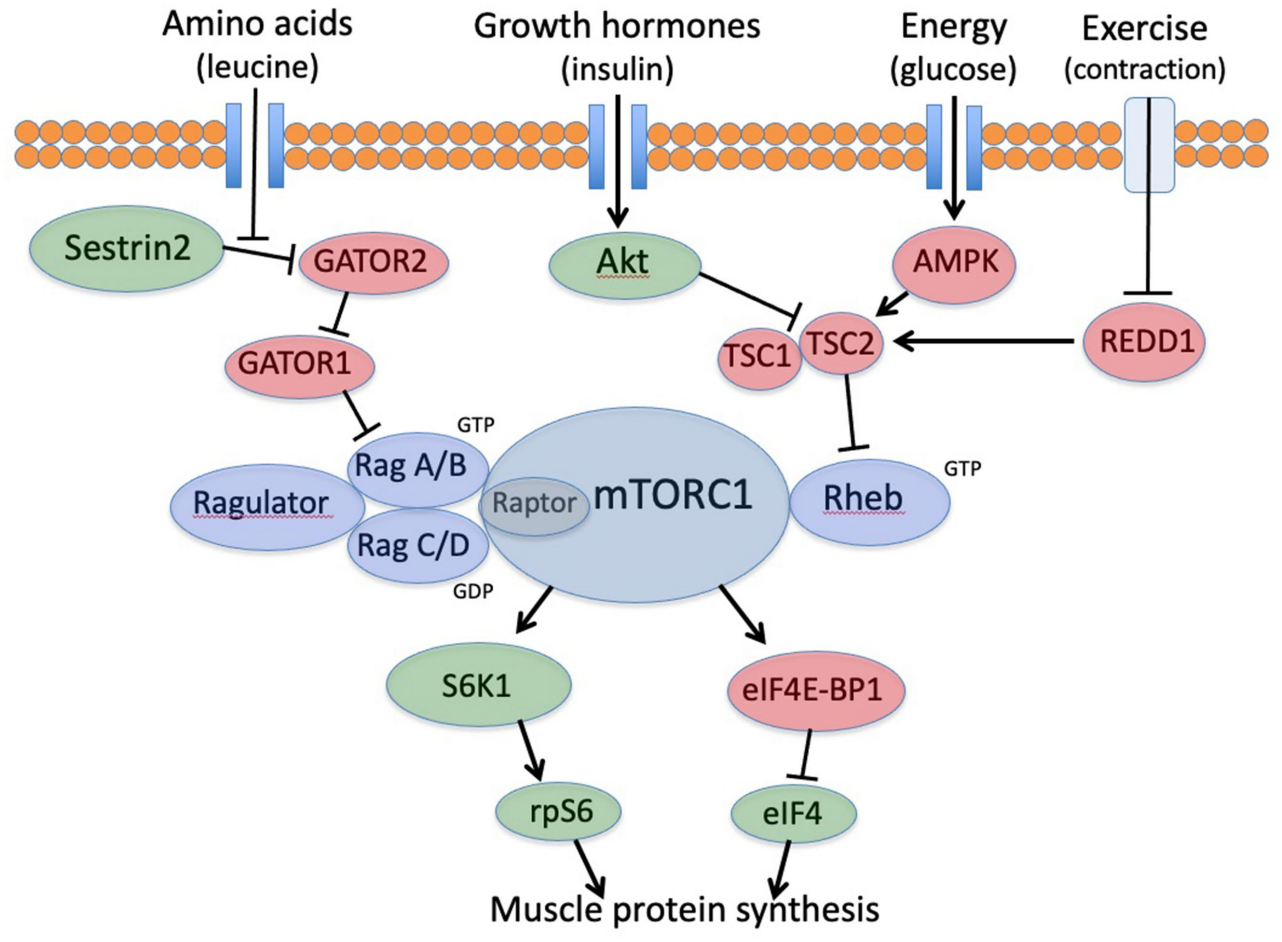
mTORC1 signaling cascade for translation initiation in skeletal muscle. mTORC1, mechanistic target of rapamycin; rpS6, ribosomal protein S6; S6K1, S6 kinase; eIF4-BP1, inhibitory binding protein complex; eIF4, active eIF4 initiation complex.

In the past 20 years, multiple laboratories have fully elucidated the leucine-mTORC1-eIF4 regulatory mechanism (Figure 1) (12, 13). The mTORC1 regulation is sensitive to multiple metabolic inputs, including amino acids (primarily leucine), hormones (primarily insulin), energy (regulated by AMPK), and resistance exercise (regulated via REDD1 and Sestrin 2) (14–16). When these inputs are optimally balanced, mTORC1 activates the downstream factors eIF4 and rpS6 (S6 ribosomal protein) to initiate MPS. These two regulatory factors serve to enhance MPS by selecting mRNAs to increase the capacity for MPS and to specifically increase the synthesis of myofibrillar proteins (17). It is important to note that the mTORC1 regulation in skeletal muscle differs from other tissues because it is sensitive to exercise (11, 18). Furthermore, the anabolic impact of insulin in skeletal muscle declines with aging while the importance of leucine increases (11, 18, 19). Other tissues remain sensitive to insulin with no known effects of exercise (19).

### The efficiency of protein synthesis response to a meal declines with aging

The second discovery was that older adults require increased amounts of EAAs to stimulate MPS. Aging reduces metabolic efficiency. My first research project in graduate school was studying age-related changes in protein synthesis (20). We discovered that the fundamental mechanisms for protein synthesis involving ribosomes and mRNAs decreased in both capacity and efficiency with increasing age. The age-related decline in MPS reduces the capabilities for repair and remodeling of skeletal muscle and is considered a central cause of muscle atrophy and sarcopenia (21). However, the inevitability of these age-related changes began to be reevaluated during the late 1990s with the findings that infusion of EAA into older adults to produce hyperammonemia could produce a robust MPS response (22). This study demonstrated that with sufficient increases in plasma amino acid concentrations, the older adults retained a capacity similar to younger adults to stimulate MPS.

Subsequently, the research group in Galveston, TX, compared meal responses of MPS in young adults (~28 years old) versus older adults (~68 years old) (23). Both groups fasted overnight and were then provided an oral dose (i.e., breakfast meal) of 6.7 g of EAA created to mimic the composition of EAA in whey protein (~15 g of whey protein). Analyzing muscle biopsies, the young adults exhibited a significant increase in MPS, while the older adults exhibited no response from the oral dose of EAA. They repeated the experiment but enriched the EAA mixture with leucine from 1.7 g in the control group up to 2.8 g in the enriched group (24). The younger adults got no added benefit from the leucine enrichment, while the older adults exhibited a rate of MPS equivalent to the younger adults. These findings demonstrated that the age-related decline in MPS could be overcome by increasing the amount of leucine in the meal and suggested that MPS has an upper limit to a meal response.

**Figure 2:**
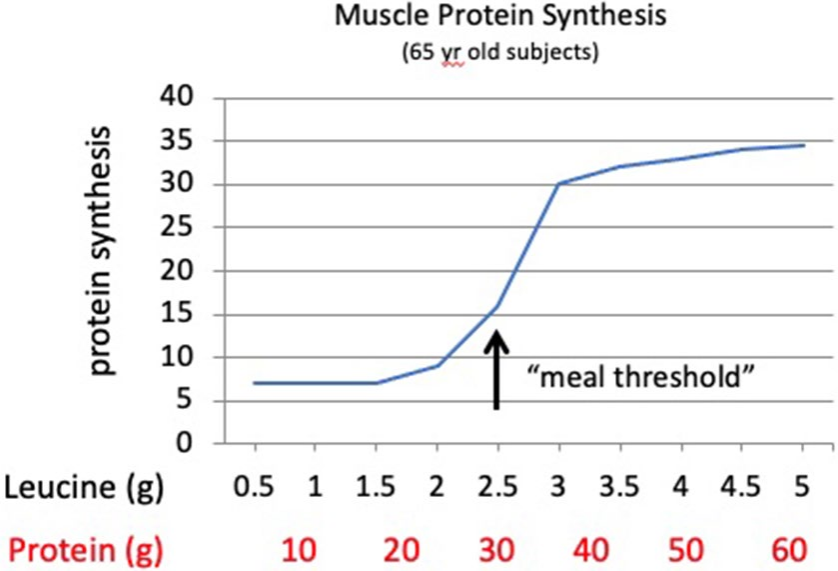
Theoretical response curve for muscle protein synthesis in older adults to increasing meal amounts of dietary protein or the amino acid leucine. Older adults demonstrate a “meal threshold” for leucine to stimulate the mTORC1 signal to initiate muscle protein synthesis. The protein amounts assume an average of ~8% leucine in meals with mixed protein sources.

The Galveston studies also highlight the important discovery that the meal effect of leucine observed in older adults is not present in younger adults. MPS in younger adults (and presumably children) appears to respond in proportion to the amount of protein in a meal. Moore et al. (25) reported that in 22-year-old males, meals containing 5, 10, or 20 g of whey protein produced a nearly linear response in MPS in proportion to the protein in the meals. Assuming the whey protein used in the meals contained ~11% leucine, the meals provided approximately 0.55, 1.1, or 2.2 g of leucine, illustrating that the leucine effect on regulating MPS observed in older adults was not evident in the young adults. Churchward-Venne et al. (26) reported a similar proportional response of MPS with 27-year-old men consuming test meals of 15 g or 30 g of milk protein. Contrary to these findings, older adults generate no meal response to 1.7 g of leucine (equivalent to ~15 g of whey protein) but demonstrate a robust response to 2.8 g of leucine (equivalent to ~26 g of whey protein) (24). These findings led to the concept of a “meal threshold” requirement for leucine to produce an anabolic response in older adults (Figure 2). A meal threshold for dietary protein and specifically leucine represents a significant modification to dietary protein recommendations (2, 3, 27). These data provide support for the theory that both the amount of protein and the EAA composition of individual meals impact the anabolic response of skeletal muscle in older adults.

While a minimum meal threshold for leucine and total protein to stimulate MPS in older adults has been established, the maximum anabolic response to protein at a meal remains controversial (3, 28, 29). Studies have shown that the MPS response after a meal follows a logarithmic pattern trending toward a plateau with decreasing efficacy of higher protein meals (25, 26, 30, 31). Moore et al. (25) found linear increases in MPS response with meals from 5 g up to 20 g of protein with no significant increase from 20 g to 40 g. Similarly, Churchward-Venne et al. (26) reported a proportional response of MPS with protein meals providing 15–30 g but no detectable difference from 30 to 45 g. Consistent with these findings, other studies have shown that meals containing 70–90 g of protein produce similar rates of MPS as meals containing 30 or 40 g of protein (32, 33). While it seems logical that there is some cellular limit to the anabolic response to a protein meal, other investigators have argued that there is no upper limit to the anabolic response to ingested protein. These investigators suggest that understanding of the anabolic response is confounded in studies of MPS because of a lack of measurement of protein breakdown (28) or because experimental designs lack sufficient duration of measurements to fully characterize the anabolic response (29). To fully characterize the optimal protein content of individual meals requires longer-term studies to establish changes in muscle mass.

An early demonstration of the impact of meal distribution was provided by the French group of Arnal et al. (34). They conducted a cross-over feeding experiment with 15 women with an average age of 68 years. The women consumed 64 g of protein daily throughout two 14-day trials. In one trial, the protein was distributed across four small relatively balanced meals (14, 20, 12, and 18 g/meal), called a spread pattern, while the other trial, known as a pulse pattern, the protein was distributed in three uneven meals (4, 51, and 20 g/meal) but with a single large meal. With the same daily intake of total protein, the pulse pattern generated higher rates of protein turnover and more positive nitrogen balance, resulting in greater fat-free mass after only 14 days. This study is consistent with a meal threshold hypothesis. Assuming that the leucine content of the meals created with a mixture of dietary proteins was ~8%, the spread pattern provided less than 1.7 g of leucine at any meal, while the pulse pattern grouped the dietary protein into a single meal providing more than 4.0 g of leucine. Similar benefits of a pulse meal pattern have been observed in hospitalized, bedrest elderly patients (35).

These findings are consistent with age-related changes in the metabolic roles of the EAA leucine. In young adults and children, leucine, along with growth hormones, contributes to the translational control of mTORC1 for MPS (18, 36), but the MPS response in young individuals appears to be proportional to the amount of protein in the meal (25). In older adults, leucine has a more specific role as a dietary signal, communicating to skeletal muscle that the meal contains adequate protein to support an MPS response (24). After a meal, activation of mTORC1 requires a twofold to threefold increase in plasma and intracellular leucine concentrations to stimulate MPS (Figures 1, 2). This metabolic role of leucine highlights the difference between the minimum dietary requirement for protein defined by the RDA versus an optimal metabolic need. The minimum leucine requirement defined by the Institute of Medicine is ~2.7 g/day for a 70-kg person (37), while the optimum amount of leucine to stimulate MPS is a minimum of 2.5 g/meal or approximately 7.5 g/day, nearly 3 times the minimum RDA (2, 3, 5).

### The anabolic response of muscle protein synthesis has a finite duration after a meal

The third important finding that supports meal distribution was the elucidation of the duration of the MPS anabolic response to a meal. When leucine meets the required meal threshold for activating mTOR and the initiation factors, it triggers MPS. The duration of this anabolic response ranges from 2 to 2.5 h after the meal (38, 39). Using whey protein, which is rapidly digested, the leucine concentration in the blood rises rapidly, stimulating MPS, which peaks at 60–90 min after the meal and declines back to the fasted baseline by ~180 min. Understanding the meal duration led to the concept of oscillating anabolic and catabolic periods for muscle protein turnover. After a meal, there is an anabolic period when MPS exceeds muscle protein breakdown, and then during post-absorptive times, there is a catabolic period when MPS declines, and protein breakdown exceeds synthesis. The catabolic period is most significant during the long overnight fast when skeletal muscle serves as a reservoir to provide amino acids to maintain essential protein turnover in vital organs.

A logical explanation for the decline in MPS after a meal would be the depletion of amino acids as they are incorporated into new protein structures. However, amino acids tend to remain elevated in the blood for 4 or 5 h or longer, depending on the amount and types of protein in the meal. However, more importantly, leucine and the regulatory proteins eIF4 and rpS6 remain elevated after MPS declines to baseline (39, 40). The limited duration response of MPS has been characterized as “muscle full” or a “refractory period” when MPS appears to be unresponsive to normal activation signals (38, 39). The underlying explanation remains speculative; however, the refractory period may be associated with declining levels of ATP to support the energy needed to maintain the elongation phase of protein synthesis (40).

The refractory period for MPS raises questions about second-meal responses. The importance of the leucine signal and the amount of protein in the first meal after an overnight fast to stimulate MPS are well-established. During catabolic periods, such as fasting or exhaustive exercise, the initiation factor eIF4 is inhibited by binding with BP1 (10, 13). This inhibition is reversed by the activation of mTORC1 and the downstream initiation proteins. While the MPS response to the first meal has been studied extensively, the MPS response to a second meal has not been studied. The findings that blood leucine and the regulatory proteins are still elevated 4 or 5 h after a first meal and after MPS returns to the fasted baseline (40, 41) suggest that the leucine threshold and eIF4 regulations may not be relevant at a second meal that occurs within 5 h after an initial stimulatory meal. Additional research is needed to characterize second meal responses and optimal dietary distribution of protein at mid-day meals.

Furthermore, the duration of the anabolic response to a meal has been recently questioned as an artifact of using rapidly digested proteins (29). These investigators suggest that consumption of 100 g of milk protein containing 80 g of slow-digesting casein can prolong the anabolic response to a meal up to at least 12 h.

Unanswered questions concerning meal duration and the oscillating pattern of protein turnover in skeletal muscle are as follows: (1) what causes MPS to decline after a meal, (2) is the observed decline an artifact of proteins selected and experimental design, and (3) is the meal response actually consistent across all meals. For example, is the first meal response after an overnight fast that inhibits translation initiation factors the same as the response to a mid-day meal when the initiation factors may still be fully active? To the best of my knowledge, there are no studies of anabolic response after a second meal (i.e., lunch), and there is some evidence that the response to protein meals late in the day is significantly lower than to the first meal (42).

In total, the available evidence from both mechanistic and clinical experiments supports that optimizing the meal response to dietary protein should be an important strategy for adults who struggle to maintain adequate protein intake and overall nutrient density while confronting declining energy needs (43). Currently, in the United States, most adults consume nearly 60% of their daily protein in a single large meal late in the day, while breakfast and the mid-day meal typically contain only 10–20 g of protein. This distribution of dietary protein fails to reach the meal threshold for leucine at either of the first two daily meals (32) and may ultimately lead to insufficient total daily protein (43).

We tested the distribution theory for impact on MPS. Using a cross-over design with 15 adult women (~37 years old), the women consumed 90 g of protein from mixed food sources (i.e., leucine content ~8%) for 7 days in either an unbalanced or balanced meal pattern (44). In the unbalanced trial, the protein was distributed as 10, 20, and 60 g at breakfast, lunch, and dinner, respectively, similar to consumption patterns in the United States. In the balanced trial, the women received 30 g of protein at each meal, designed to provide at least 2.5 g of leucine at each meal. After the first day and the seventh day, 24-h net protein synthesis was measured in skeletal muscles. While the women consumed the same total protein each day, the balanced meal distribution produced greater net 24-h MPS than the unbalanced distribution.

## Evidence that protein distribution at meals impacts body composition

While the application of the meal threshold hypothesis has been tested by redistributing protein from dinner to the first meal to enhance MPS (44), the long-term effects on body composition and muscle mass remain unclear. There are studies reporting the benefits of meal distribution of protein for body composition and muscle mass (44–46), while other studies fail to find significant effects (47). The inconsistency of the findings may, at least in part, be explained by considering the likely magnitude of body composition changes during short-term studies, which are likely within the detection limits considering variations among subjects and current body composition methods.

### Meal distribution of dietary protein impacts body composition in animals

To test the meal distribution hypothesis and estimate the magnitude of the body composition effects, we designed a meal distribution study with adult rats (48). Rats were trained to consume meals similar to the meal pattern used in our human MPS study (44), with their daily ration partitioned at meals providing 4, 4, and 6 g of food. One group of rats received protein in a balanced pattern with 16% of energy (%En) from protein at each meal, while the other group received an unbalanced distribution of 8%En, 8%En, and 27%En, respectively. The total daily diets for both groups were exactly the same for calories, protein, carbohydrates, fat, and fiber. The only difference was the distribution of the protein and carbohydrates. The design was built around both protein and leucine distributions. Previous studies (28) identified the meal threshold for leucine with this age and size of adult rats as 55–60 mg. With the balanced distribution, the meals provided 74, 74, and 111 mg of leucine, and in the unbalanced distribution, the meals provided 38, 38, and 184 mg. With the balanced distribution, all three meals provided sufficient leucine to activate MPS, but with the unbalanced distribution, only the last meal exceeded the leucine threshold for activation of MPS.

After 2 and 11 weeks, MPS was determined after the first meal, and eIF4, rpS6, and MPS were found to be 30 to 45% higher in the animals consuming the higher leucine meal. Body composition was measured by DEXA at 11 weeks. Surprisingly, there were no significant differences in fat mass or fat-free mass between the groups, suggesting the meal distribution had no effects. However, direct dissection of tissues revealed that the hindlimb muscle mass was ~10% larger in the animals with the balanced distribution, while the liver was ~10% larger in animals receiving the unbalanced distribution with the large dinner meal (48). These findings are consistent with the leucine threshold hypothesis for MPS and also demonstrate that whole-body DEXA measurements do not differentiate small, tissue-specific changes in lean body mass.

### Meal distribution of protein impacts body composition during weight loss

Recognizing that meal distribution likely has a small impact on muscle mass and is likely secondary to protein quantity and quality, definitive proof for benefits related to aging and sarcopenia that are characterized by changes of only 5–8% per decade will be difficult to obtain. An alternative approach is to apply meal distribution concepts during weight loss when body weight is changing more rapidly, and lean body mass can account for up to 50% of the total weight lost.

We applied the leucine threshold and meal concepts to a series of weight-loss studies (49, 50). These studies modified both the quantity and the meal distribution while protein quality remained similar across treatment groups. In each of the studies, the diet design was the same, and the daily energy restriction was approximately 500 kcal from their pre-study diet. Participants were randomly assigned to either a high carbohydrate, low protein diet (55%En carbohydrates, 30%En fat, 15%En protein; 0.8 g protein/kg body weight) with meals providing 10, 15, and 45 g of protein, respectively, or to a reduced carbohydrate, higher protein diet (40%En carbohydrates, 30%En fat, 30%En protein; 1.6 g/kg) with protein distributed as 35, 35, and 50 g. While higher protein at the first meal has been shown to enhance appetite regulation (satiety) and thermogenesis, the hypothesis for these studies was that increasing protein at the first meal would enhance MPS, minimizing loss of lean body mass and resulting in greater loss of body fat.

In the 12-month diet study, 130 overweight men and women (BMI ~33; age ~ 45 years) were randomly assigned to either the low-protein or high-protein diet groups (49). The average weight loss at 12 months was 24% greater in the higher protein group with significantly greater loss of body fat (5.3 kg vs. 7.3 kg, in low- and high-protein groups, respectively). Loss of lean body mass (LBM) was similar (2.7 kg vs. 2.6 kg, respectively); however, the net change in body composition was significantly different, with LBM accounting for 34% of the weight loss in the low-protein group and 26% in the higher protein group.

Similarly, in a weight loss study conducted with community-dwelling older adults (~70 years old), participants who voluntarily shifted daily protein intake from dinner to earlier meals lost more total weight and more body fat without changing total daily protein intake (51). The researchers concluded that “a more even pattern of protein intake was associated with a greater decline in BMI and abdominal fat”.

In a second study utilizing the same diet protocol, we evaluated the additive and synergistic effects of dietary protein and resistance exercise on body composition changes during weight loss (50). Utilizing a 2 × 2 design, 48 women (BMI ~33; age ~ 46 years) were randomly assigned to one of four treatment groups: low protein, low protein with exercise, higher protein, and higher protein with exercise. Similar to the previous study, the dinner meals were similar across all groups. The primary diet differences were increased protein and reduced carbohydrates at the first two meals in the higher protein groups. After 16 weeks, the higher protein (diet only) group lost 12% more body weight, 18% more body fat, and 25% less lean body mass compared to the low protein group. Consistent with the previous study, 35% of the weight lost for the low protein group was fat-free mass, and 25% for the higher protein group.

The exercise treatment consisted of 5 days/week of walking for 30 min and 2 days/week of resistance exercise (49). After 16 weeks, the higher protein + exercise group lost 46% more body weight, 60% more body fat, and 40% less fat-free mass compared with the low protein + exercise group. This study demonstrated the synergistic effects of dietary protein and exercise to improve body composition during energy restriction for weight loss. Furthermore, the addition of 16 weeks of exercise to the low protein treatment group resulted in the loss of an additional 0.5 kg of body fat compared with the low protein group without exercise, while the addition of exercise to the higher protein group resulted in the loss of an additional 2.9 kg of body fat compared to the diet group without exercise. To the best of our knowledge, this was the first study to demonstrate the interactive effect of dietary protein and exercise on improving body composition in adult women during weight loss.

While these weight loss studies appear to demonstrate the benefits of increased protein at the first meal, the studies do not differentiate effects due to increasing daily quantity versus meal distribution. However, the studies build on the discoveries that increasing dietary protein at the first meal stimulates MPS and increases net MPS for the day. The assumption inherent to this design was that adding 50 g of additional protein to a dinner meal that already contained ~50 g of protein would have a minimal additive effect on net daily MPS (25, 26, 32) or muscle mass (45).

### Population survey support for meal distribution of protein

Population studies, in general, have not focused on meal distribution of protein, and the quality of information on meal-specific protein distribution is limited in most food surveys. The NHANES data reveal that higher daily protein intake is inversely correlated with BMI and waist circumference (52), and the findings appear to be associated with increased protein at breakfast (46, 47). Again, meal distribution is often intertwined with total protein intake. Studies using NHANES data show that adults consuming 2 or 3 meals with at least 25 g of protein at each meal are more likely to meet the minimum RDA for protein (43) and maintain greater muscle mass (44, 46). Kim et al. (47) reported adults who consume a greater percentage of their total daily protein at breakfast maintained greater muscle mass and grip strength than individuals consuming a high percentage at the dinner meal. These same investigators also conducted an intervention study and found that supplementing 30 g of protein at the breakfast meal with older adults produced greater muscle mass than supplementing 30 g of protein at the dinner meal. Overall, while the number of studies is limited, population-based surveys appear to support the merit of multiple protein meals per day, with increased protein at the breakfast meal providing additional value.

## Summary and conclusion

In summary, the direct effects of meal distribution of dietary protein on muscle mass in older adults are difficult to assess. Changes in mass occur slowly and are likely small in magnitude, and methods for directly measuring muscle mass are limited. There is a general assumption that short-term measurements of MPS provide a biomarker for anabolic changes in muscle mass; however, changes in MPS are of much greater magnitude than changes in muscle mass (53). Still, there are some fundamental metabolic responses that support meal distribution. The first is the discovery of the meal threshold for leucine to trigger MPS and the related discovery of the duration of the post-meal anabolic response. Triggering the mTOR signal complex to initiate MPS requires approximately 3.0 g of leucine, which is equivalent to a meal containing approximately 30–35 g of high-quality protein, and once activated, MPS will remain elevated for approximately 2.5 h. Adding more protein to a meal does not increase the magnitude or duration of the anabolic period (25, 26). The logical extension of these findings is that adding protein to a low-protein meal would be more beneficial than adding protein to an existing meal already containing maximum protein for MPS effects. Furthermore, there is a general belief that MPS is most responsive at the first meal after an overnight fasting period. Essentially, every study of MPS in either humans or animals has been done at the first meal, maximizing the recovery of translation initiation factors inhibited during the overnight fast. If MPS measured at the first meal is not a relevant biomarker for anabolic changes in muscle mass, then the significance of studies measuring MPS after this first meal must be re-evaluated.

Furthermore, evidence accumulates that protein quantity and meal distribution are interrelated in protecting adult muscle mass. The first priority is achieving a single meal with adequate protein and leucine to stimulate MPS (26). If the daily protein intake is limited to the RDA of 0.8 g/day (~60 g/day), the daily protein intake needs to be aggregated into at least one meal with >35 g of protein. Evenly distributing the low protein intake across multiple meals with <20 g of protein minimizes MPS responses and the benefits to skeletal muscle. However, if protein intake is higher (~1.6 g/kg; 120 g/day), adding additional protein to large dinner meals that may already provide >50 g of protein is likely inefficient for muscle benefits. Research demonstrates that adding protein to the first meal enhances MPS and produces benefits to muscle mass and body composition (46–51). The application of these findings and the meal distribution hypothesis to long-term muscle health, such as aging and sarcopenia, remains difficult to prove and awaits additional research.

### Recommendations

Based on the weight of available evidence, we believe that older adults benefit from daily protein intakes above the RDA ranging from 1.2 to 1.6 g/kg (27). Furthermore, the evidence supporting the anabolic response at the first meal is robust, and we strongly recommend increasing protein intake at breakfast to at least 30 g of high-quality protein (2, 3). The optimal distribution of dietary protein across all meals requires additional research and an integrated understanding of the interrelationships of dietary protein quantity, quality, and meal distribution with age and physical activity.

## Author contributions

DL: Writing – original draft, Writing – review & editing.
